# Bristol Medico-Chirurgical Society

**Published:** 1899-03

**Authors:** 


### MEETINGS OF SOCIETIES.

### Bristol Medico-Chirurgical Society.

*December 14th*, 1898.

Dr. R. ROXBURGH, President, in the Chair.

Dr. J. MICHELL CLARKE showed a man, aged 60, suffering from acromegaly. The characteristic enlargement of the lower part of the face, hands and feet was well marked. The tongue was very large, and the speech consequently indistinct. The back was bowed, the sternum and chest massive, and the head carried forwards. With the exception of slight deafness, the special senses were normal, and the visual fields were of normal extent. The fundus oculi was normal in each eye. The patient said that neither he nor any of his friends had noticed any change in the appearance of his face or hands, and he thought that his appearance had been as it is now for many years. He had always enjoyed good health, and been able to follow a laborious occupation, and complained only of pains in the loins of two weeks' duration. He had never had syphilis.

Dr. C. ELLIOTT showed a boy, aged 11 years, suffering from a skin eruption which seemed to conform to Hebra's "lichen ruber planus." The disease had existed for over five years, and had begun after an attack of scarlatina for which he had been treated in the Homerton Fever Hospital. The patches, at first the size of millet seeds, have gradually enlarged till now they vary from the size of a threepenny piece to a crown-piece. They are situated on the trunk, both upper and lower extremities, under the chin, on the forehead, and both ears. There is no history of syphilis, but the boy had suffered from hip-disease on the left side about six years. His general health is now good, and he has no trouble with his hip beyond some shortening. Both parents are living and well. Mother has had seven children: four (including the patient) are living; two died of diphtheria, and one was still-born. All treatment had been of little or no benefit. Arsenic, iron, mercury with iodide of potassium, and thyroid extract had been given internally.

Locally, ointments of ichthyol, resorcin, chrysophanic acid and borax, the latter seeming to be the most useful. In the autumn, scarification was tried—"mincemeating" the patches; and although these had not been cured, yet a good effect had been produced, namely, in relieving the constriction felt by the patient and allowing him greater freedom of movement.—Dr. H. WALDO thought it may be a case of what Jonathan Hutchinson calls lichen-psoriasis; but unfortunately he could not discover any papules, the presence of which would make the diagnosis easy. There were only patches accompanied with, perhaps, some pigmentation.—Dr. A. J. HARRISON expressed his doubts about the case being one of lichen ruber planus; but it might be one of the rare forms described by the late Professor Hebra. The papular character is very characteristic in these cases, the angular nature of the papules being well marked. Dr. Harrison showed a photograph of a very remarkable case of the disease.

Dr. E. C. WILLIAMS showed three patients: (1) A case which had been exhibited before the Society[1] thirteen months ago, treated by hot air. After twenty-five baths the boy was able to walk; and now, after the lapse of thirteen months, he is able to walk without difficulty and has no limp. (2) A woman who had rheumatoid arthritis for twenty years. She was unable to dress herself or use her needle. She also had a rash, like psoriasis. She has had forty baths, and the joints are now quite well and the rash has also disappeared. She has had no joint-trouble for the last three months. (3) A child who had had patches of baldness for six months before she came to the Children's Hospital. She was treated with blisters, and also with thyroid extract, without any improvement. For the last five months she has been treated with the oxygen cap, and the hair has grown rapidly. The cure may have been a coincidence.—Dr. B. M. H. ROGERS said that the first case was under his care for some time. The improvement was very considerable.—Dr. J. SWAIN did not think that the boy's stiff knee could be called rheumatoid or tubercular. It was most likely traumatic.—Mr. PAUL BUSH was inclined to think that this case might be looked upon as a cure of a stiff knee by movement.—Referring to Dr. Williams's case of alopecia areata, the PRESIDENT advocated the use of pilocarpine internally in this affection, $\frac{1}{16}$th gr. three times a day, together with the local application of the same alkaloid and the weekly shaving of the affected spots. He had seen excellent results from this treatment.—Dr. E. J. CAVE considered it difficult to form an opinion as to the efficacy of treatment in case of alopecia areata, as so many of the cases get quite well under the most various treatment or none at all. He had seen some cases lately which have got well under the local application of formalin.—Dr. C. ELLIOTT could not agree with Dr. Cave that alopecia areata got well without any treatment, although he believed that cases got well under very varied treatment.—Dr. WALDO had found cases of alopecia areata do well with the oxygen cap, but they seem to do equally well with the cap without the oxygen. Saboraud has shown that the disease is caused by a specific bacillus which corresponds to that of the seborrhœa micro-organism.—Dr. E. C. WILLIAMS did not think the knee was tubercular, nor was he prepared to say it was rheumatoid; there was an indefinite history of rheumatism and a history of an injury.

Mr. C. A. MORTON showed a specimen of a large malignant tumour of the kidney, removed by abdominal nephrectomy from a child eighteen months old. It was one of those complex malignant growths

[1] *Bristol M.-Chir. J.*, 1897, xv. 363.

of the kidney which occur in young children—a mixture of sarcoma and new gland-tissue. The upper part of the kidney was not invaded by the growth, but the lower part had been replaced by it. The tumour occupied half the child's abdomen, and projected considerably. In July last, the child was taken to Dr. Cotton for a discharge of blood which was found running down the thighs, but the source of the bleeding was not clear at first. It then became evident that occasional hæmaturia was present, and the abdominal swelling was discovered. Mr. Morton saw the child with Dr. Cotton on August 4th, and operated on the 18th. In the removal of the growth, the posterior layer of the meso-colon was divided, and then the colon drawn forwards. The ureter was divided between double ligatures, but the vessels of the kidney were too much on the stretch to be safely tied, and had to be clamped before division and ligated after. A separate incision was made for drainage in the loin. The amount of shock was moderate, and soon passed off. There were no symptoms after the operation, and primary union took place. At the end of November Dr. Cotton reported that the child's health was not good, but there were no signs of recurrence. Mr. Morton thought that those members of the Society who had perhaps noticed his remarks in the June number of the *Bristol Medico-Chirurgical Journal* might be surprised that he had performed the operation: he had not recommended it, but had placed before the parents the great risk of operation, and the great chance of recurrence; and they had decided to take the risk for the chance of prolongation of life or the very remote one of cure,—and they had been fortunate in doing so, for at any rate the child's life would be prolonged by several months even if recurrence took place at an early date.—Mr. ROGER WILLIAMS said that hitherto results of such operations had been most unfavourable. Several years ago Newman's statistics[1] showed that three-fourths of those operated on died from the effects of the operation, and of the remaining one-fourth all were dead within a year. A later series showed mortality from the operation of two-thirds, and the after-results were no better than in the foregoing series. Recently, Walker's cases[2] showed mortality from the operation of rather less than one-half, and of the remainder several had survived free from recurrence for upwards of three years. It would require considerable research to unearth, out of the several hundred operated cases on record, a score of "cures" who had survived the operation upwards of three years. This mortality was due to great malignancy of these infantile sarcomata, which exceeded that of corresponding disease in adults; frequency with which disease is bilateral (50 per cent.), and multiple *ab initio*. Mr. Williams thought that some of these tumours were more malignant than others, the striped-muscle-containing tumours being more malignant than the adeno-sarcomata; and he remarked on the peculiarity that these two types of sarcoma seldom co-existed, which suggests probable difference of origin. He thought that the best results hitherto attained had been in dealing with the adeno-sarcomata. Renal sarcoma being the commonest form of infantile malignant disease, these cases were of much interest, especially to surgeons.—Mr. T. CARWARDINE suggested the possible origin of the tumour from remains of the Wolffian body, which have been found in the fœtus at the lower and anterior part of the kidney—identical with the situation of this tumour, which appeared rather to invade the kidney secondarily.—In reply to Mr. Roger Williams, Mr. MORTON said the work of Kelynack on *Renal Growths*, and the very important paper by Walker, of the Johns Hopkins

[1] *Diseases of the Kidneys*, 1888. [2] *Ann. Surg.*, 1897, xxvi. 529.

Hospital, of which he had given an abstract in the June number of this *Journal*, gave the most recent information as to the mortality after operation. The one encouraging fact was that it was diminishing. He could not agree with Mr. Williams that there were even a score of cases which had lived for three years after nephrectomy and therefore would be called "cures" according to Volkmann's law. There were two such cases operated on by one surgeon — Abbe, of New York, and two more by German surgeons, and he believed those were all the cases recorded as alive three years after operation. With regard to the bilateral nature of the disease, he called attention to the fact that in the 145 records of cases collected by Walker, the bilateral character of the growth was not marked, though the general view was that it was usually bilateral. Mr. Morton could not agree with Mr. Carwardine that the tumour had arisen outside the kidney. He thought that the fact that the whole organ had not been destroyed by the growth was no reason for rejecting the view that it was essentially a renal growth. A large part of the kidney had been replaced by the tumour. These renal growths of early life had by several pathologists been regarded as arising in remains of the Wolffian body, and the gland tissue when present was thought to resemble more that of the Wolffian body than the kidney.

Dr. James Swain showed a specimen of double pyo-salpinx removed from a married but nulliparous woman, who had had severe pelvic pain for two years. The specimen was a good example of large pyo-salpinx, which formed a distinct abdominal tumour—a circumstance which is more infrequent than usual. The operation presented no special difficulty beyond the adhesions which usually exist in these cases, and the patient made an uninterrupted recovery. The pus in such cases is frequently sterile, and that was so in the present case.

Dr. J. O. Symes showed microscopic specimen and lantern slide of tetanus bacilli in pus. The film was prepared from a suppurating wound of the hand in a patient under the care of Mr. Harsant, at the Bristol Royal Infirmary. Death resulted from tetanus, and cultures of the bacillus were obtained from the wound after death. The number and size of the bacilli were unusually large. Many pyogenic organisms were also present.

Dr. Michell Clarke showed stained blood-films, and lantern slides which showed the various stages of the malaria parasite—tertian variety.

Mr. G. Munro Smith exhibited a series of photo-micrographic lantern slides, prepared by Mr. J. Taylor, illustrating the pathology of rodent ulcer, adenoma of the skin, tubercular ulcer of the tongue, myeloid sarcoma and rickets.—Mr. C. A. Morton had examined many sections of rodent ulcers, and thoroughly agreed with Mr. Munro Smith that there was not a downgrowth of surface epithelioma, and that they were histologically as well as clinically well differentiated from epithelioma. He objected to the term malignant adenoma which Mr. Smith suggested for rodent ulcer, the type of malignant adenoma of the form of cancer which occurs in the intestine; but here there was a reproduction of gland tissue, whereas in rodent ulcer we found only irregular masses of epithelial cells, and not gland tissue at all. He could not accept Mr. Munro Smith's view that the malignancy of epithelioma consisted in the small round-celled proliferation. He thought the essential condition of malignancy in epithelioma was the invasion of the deeper tissues by the advancing columns of epithelial cells, and he regarded the small round-celled growth as inflammatory, and due to the irritation of the epithelial invasion. He was glad Mr. Smith had shown the slide of tissue removed from the edge of an ulcer of the tongue for purposes of diagnosis, for he had again and again proved the great value of this method in the diagnosis of ulcers on the tongue of doubtful nature. It was much better than taking a mere scraping or than wasting time in giving iodide of potassium. Only cocaine was necessary as an anæsthetic.—Mr. ROGER WILLIAMS thought it could not be maintained that rodent ulcer was exclusively of sebaceous gland origin. It was quite true that hypertrophied sebaceous structures were often concomitant with rodent ulcer; but these were seldom cancerous. The examination of a large number of specimens had convinced him that the disease occasionally arose from the appendicular structures—hair follicles, sweat glands, and sebaceous glands; but he believed that most cases arose from the interpapillary processes of the *rete*, independently of the glands, etc. From the histogenetic standpoint these were different types of rodent ulcer. That the appendicular structures were not essential to the origin of the disease was, he thought, proved by the fact that rodent ulcer sometimes arose from the dermo-papillary membrane lining the *portio vaginalis uteri*, which was skin, without any appendicular structures (sebaceous glands, sweat glands, hair follicles, etc.). He had met with a case of this kind, in which the disease evidently started in the *rete;* and this he believed was its usual origin.—Dr. F. H. EDGEWORTH remarked that the generalisation drawn by Mr. Munro Smith from his section of rickets was probably too wide, as at the last meeting of the Bath and Bristol Branch of the British Medical Association Mr. Flemming had shown sections of rickets in which these appearances were not present.[1]—Dr. MICHELL CLARKE said that he had seen as irregularly-shaped cartilage-cells as those shown on the screen in the specimen of rickets in the normal development of cartilage into bone.

---

*January 11th*, 1899.

Dr. R. ROXBURGH, President, in the Chair.

Mr. C. E. S. FLEMMING showed microscopical sections of an angeio-sarcoma from a woman aged 68, who two years before her death fell and hurt her back. A week after the accident she had pain in her sacrum; this continued for nine months before any direct evidence of a growth could be discovered. There was then a pulsating tumour over the right sacro-iliac synchondrosis, with a loud bruit heard down to the femoral artery. The tumour increased steadily in size, latterly causing paralysis of the bladder and pain in the right leg. There was no clinical evidence of any secondary growth. By a partial *post-mortem* examination nearly the whole of the sacrum and a considerable part of the right ilium were found to be destroyed by a large soft mass in appearance like organising bloodclot. Under the microscope this growth was found to consist almost entirely of embryonic blood-vessels, with intervening small collections of nucleated round cells and a few laryngo-myeloid cells. The cells of the blood-vessels were in places markedly columnar, and between the different vessels there could be distinguished in places a fine fibrillar matrix. It appeared probable that the growth might have originated in the cauda equina.—Mr. ROGER WILLIAMS said that though this form of neoplasm was rare, it had now been demonstrated in most parts of the body. If we accept the view that it arose from the peri-epithelium or endothelium of the blood-vessels and lymphatics there was still some difficulty in deciding whether it should be classed with the sarcomata or with carcinomata, because embryologists were at variance as to whether endothelial structures were of archiblastic or of parablastic origin. In their clinical character growths of this kind

[1] *Vide* p. 21 of present number.

generally resembled carcinomata, especially in that they were very prone to disseminate, and he enquired if there were any signs of malignancy in this case. He thought growths of this kind were interesting in connection with Ackermann's hypothesis of sarcomatosis, according to which all sarcomata arose in the immediate vicinity of blood-vessels or lymphatics, every tumour consisting of interwoven neoplastic fasciculi with a central blood-vessel.

Dr. WALDO read a paper on hemorrhoids, which, he said, were always pathological. By far the greater portion of the blood from the plexus of hemorrhoidal veins is carried into the inferior mesenteric vein, and thence into the vena portæ, through the medium of the superior hemorrhoidal vein. The circulation through the portal system is subject to much interference in consequence of hepatic and intestinal obstruction, and in these changes the blood in the hemorrhoidal plexus also participates; and were it not for the provision that exists by which this plexus may free itself to a certain extent from over-distension by its communication with the internal iliac vein, through the medium of the middle hemorrhoidal vein, piles would be much more frequent than they even now are, as a consequence of obstructed portal circulation. All these veins are valveless, and are therefore liable to over-distension whenever the abdominal circulation is sluggish. An invariable accompaniment of internal piles is a greater or less degree of prolapse of the mucous membrane of the rectum, and chiefly on this account the patient should be instructed to have his daily motion at bedtime. The chief reflex phenomena encountered are a dull aching fixed pain at the lower part of the lumbar spine, which may extend down the thighs or round the groins, and irritation in the neighbouring organs. Patients should adopt a rational way of living. The diet should be so regulated as to leave behind the least possible solid residue. Highly seasoned food, or vegetables containing much cellulose and the leguminous vegetables, should be avoided. Meat or fish, rather than much starchy food, is advised. And green vegetables with stewed fruits and preserves, with a fair quantity of diluents, assist the daily action of the bowels. Fat of meat, or cream or butter, may be taken, as oleaginous substances are found to assist the flow of the bile and so indirectly the vascular circulation. Moderate exercise should be taken in various ways. The tendency to bleed after fifty years of age gradually grows less and finally disappears. The tumours may remain, and with them the old tendency to prolapse; but generally they atrophy, leaving little if any future discomfort unless the prolapse is bulky. Treatment by drugs was touched upon, and a mechanical mode of treatment upon physiological principles was explained and advocated. He believed that physicians could assist these patients immensely; but in some cases, whether medical treatment is neglected or not, the only right proceeding is to hand them over to the operating surgeon. A young clergyman from South Africa, who consulted Dr. Waldo a few months ago, told him that two-thirds of the clergy and doctors in that part of the world were operated upon for piles (some of them two or three times), the cause of this being that they averaged fifty miles a day in the saddle.—Mr. MUNRO SMITH suggested that the liver had not so much to do with hemorrhoids as was usually thought. He doubted if obstruction in the portal circulation occurred apart from definite disease of the organ, and if it occurred whether it could be diagnosed.—Dr. AUST LAWRENCE referred to the necessity in all cases of ascertaining by a digital examination that piles really did exist; he mentioned several cases that he had seen treated for piles where the disease was of a malignant character, and often in women all the symptoms were due to uterine displacement.—Mr. C. E. S. FLEMMING

objected to the use of glycerine injections, because of their liability to cause painful and troublesome tenesmus and occasionally hemorrhage. He also wished to know in which class of cases anal gymnastics should be adopted, as stretching of the sphincter had been frequently recommended as a method of treatment.—Mr. Ewens had seen many cases of *supposed* piles, which proved to be due either to retroflexed or retroverted uterus, or to an enormous accumulation of fæces in the rectum. Appropriate treatment relieved these cases. He had met with cases of supposed piles which were really malignant disease of the rectum. He had seen much benefit from the administration of hazeline in bleeding piles in connection with mild aperients.—Dr. Harrison suggested that hazeline has been long known as efficacious in the treatment of hemorrhoids, invariably as a well-known American popular remedy—Pond's Extract—containing hazeline. Dr. Harrison was surprised to hear that riding on horseback tended to produce piles: of course much exercise would not be advisable where piles were actively asserting themselves, but as a preventative to liver congestion and hemorrhoids the late Lord Palmerston's dictum was a very wise one; viz., "that the outside of a horse was the best thing for the inside of a man." Dr. Harrison also controverted Dr. Waldo's suggestion that pruritus ani was common with piles, Dr. Harrison associating it much more with eczema and fissure of the anus.—Dr. Watson Williams considered that while in the less pronounced cases of hemorrhoids medical treatment might be attended with very favourable results, it was well to bear in mind the limitations of medical methods, and in suitable cases to call in the aid of a surgical colleague. He did not think that the advantages of a radical surgical cure were sufficiently often impressed on patients, whose lives were not seldom marred by a condition that could be, and should be, completely removed. —Mr. Carwardine pointed out that piles have recently been shown by dissection to be really cavernous angeiomata, fed by the superior hemorrhoidal artery, and consequently not of venous origin,—Dr. Elliott considered heredity an important factor in the causation of piles. He believed bicycling and riding on horseback were of decided benefit to this class of patients. With regard to operation, he begged to remind members that the patient as well as the surgeon had to have a voice in the matter; and when the former heard of death following an operation among his friends, he was not surprised that patients welcomed other modes of treatment. Personally he was acquainted with two fatal cases due to secondary hemorrhage following operation; and knew of a third case, where a fatal result was fortunately prevented by plugging the rectum. Such cases, of course, did not appear in our medical journals.—In reply, Dr. Waldo said, as a large portion of the blood is obliged to return to the heart through the liver, hemorrhoids would certainly be influenced by any obstruction occurring in the latter organ.

Dr. Aust Lawrence read a paper in which he urged (1) the necessity of repairing all perineal injuries after labour; (2) the use of catgut with which to repair these injuries, and the non-inclusion of the skin in the operation; (3) the importance of keeping on the forceps until the head is delivered, as this often prevents and ought never to cause rupture of the perineum; (4) the wisdom of telling the husband, etc., that with the use of forceps there may be laceration of the perineum; but it is not caused by the forceps, but by the large size of the child's head and by the small parts of the mother.—Dr. Firth mentioned a case in which he had been asked to repair a perineum seven days after confinement.

He had succeeded in persuading the patient to have the operation postponed for at least six weeks. Dr. Firth asked Dr. Lawrence if he would consent to operate seven days after confinement, and if not, how long he would wait.—Dr. Newnham said that it is perhaps well, in putting sutures into the perineum immediately after the confinement, to be careful not to sew up the rectum entirely. A case sent to him lately had the perineum and rectum so completely sewn up that ntestinal obstruction was caused; and, finally, an anus was formed in the posterior wall of the vagina. It is, therefore, important to see that a direct outlet is left for the fæces after sewing up a bad rupture of the perineum.—Dr. Walter Swayne said that a most important point with regard to the immediate union of lacerations of the genital tract was the prevention of septic poisoning. In his experience of puerperal septicæmia the most hopeless and intractable cases were those in which lacerations of the vagina and perineum were present. The raw lacerated surface allows of direct infection of the lymphatic system by septic micro-organisms, and the production of a most acute form of septicæmia. Then again minor forms of septic infection, in which the general symptoms were more urgent, led to the production of sub-involution of the uterine body and ligaments, and in this way predisposed to displacements such as procidentia. On these grounds alone the immediate union of all lacerations should be the invariable practice. He would not criticise Dr. Lawrence's paper, as his remarks should meet with universal acceptance.

Dr. J. Lacy Firth read a paper on a case of primary sarcoma of the thyroid gland, in which a marked amelioration of symptoms for four months had resulted from partial thyroidectomy, the right lobe having been removed. The rarity of this form of growth was alluded to, and the almost uniformly rapid fatal result of operations upon them.—Mr. Harsant referred to a case of primary carcinoma of the thyroid body, upon which he had operated for the relief of dyspnœa; the patient only survived a short period. He had never met with a case of sarcoma of the thyroid.—Mr. Roger Williams said he had made an analysis ot all the primary tumours under treatment at four large Metropolitan hospitals during a period of from ten to fifteen years. Of 7,294 cancers only seven originated in the thyroid, and of 1,266 sarcomas only one started in the thyroid. He knew of several instances of malignant thyroid neoplasms arising in early life. Zahn had reported an instance in a fœtus. In several cases chondromatous, rhabdomyomatous and squamous epithelial structures had been found in these tumours; and he asked if heterotopic elements of this kind had been noted in Dr. Firth's specimen-—Dr. Stack asked whether there was any diminution in the portion of the gland left, as it is said that sometimes malignant disease of the thyroid for a time diminishes after partial operations.—Dr. Waldo asked whether Mr. Firth had administered thyroid extract to patients with non-malignant enlargement of the thyroid gland, considering that the current view was that the system called upon the thyroid body for an increased supply of its excretion, and that the gland responded and as a consequence hypertrophied. It is said that if the extract is given by the mouth in some of these cases the thyroid body diminishes in size, and the patient recovers.—Mr. Morton could not quite agree with Mr. Harsant that removal of a portion of the gland was the only method of dealing with urgent dyspnœa in these cases. He thought an opening into the trachea above the isthmus, and the passage of a large tube such as a wide gum-elastic catheter below the obstruction, might be done in desperate cases.—Mr. Paul Bush remarked that in two of his cases of malignant disease of the thyroid gland, in which a partial removal only could be performed, there had been a considerable diminution in the size of the growth following operation, lasting in one case for several months: the growth in this case was undoubtedly a sarcoma; both patients, however, died of the disease eventually.—In reply, Dr. J. LACY FIRTH remarked that microscopical examination showed his case to be one of pure sarcoma of the thyroid. Here and there in the section remains of the thyroid vesicles undergoing destruction were to be seen. In the published drawings of secondary thyroid tumours found in bone and other tissues, there was no appearance of sarcomatous tissue, and they were, therefore, neither sarcomata nor adeno-sarcomata. In his case there had been a very noticeable diminution of the left lobe of the gland for a time after the operation of thyroidectomy on the right side. He had tried the effect of thyroid extract in two cases of enlarged thyroid. The first case was one of sporadic cretinism, and the thyroid gland was the largest he had seen and of the adenomatous type. The extract rapidly produced a marvellous diminution in the size of the gland and amelioration of the symptoms. In the second case, one of ordinary goitre, the effect of the medicine had been favourable, but not to a marked degree.

---

*February 8th*, 1899.

Dr. R. ROXBURGH, President, in the chair.

Dr. F. H. EDGEWORTH showed a specimen of abscess of the brain. The patient, a woman aged 43, who had never had any previous illness, was on December 2nd seized with motor aphasia, and paralysis of the right side of the face and tongue and of the right arm, without loss of consciousness. Ten days later the right leg became paralysed and the intelligence blunted, and a week subsequently she died with symptoms of pressure on the left cerebral hemisphere. No lesion or abnormality of the vascular system was detected, and the urine was normal. The *post-mortem* examination showed an old abscess of the left nucleus lenticularis, which had recently invaded the left internal capsule. The rest of the body was intact. The abscess, from its situation, must have been embolic in origin, but no source of septic embolism was discovered. Gross organic lesions of the brain, such as tumour and abscess, may in some instances give rise to suddenly developing symptoms; but there is usually some evidence, such as headache, vomiting, or optic neuritis, which suggests that the lesion is other than a vascular one. Such evidence was wanting in this case.

Dr. SYMES read a paper on "The Bacteriology of some Suppurations complicating Pulmonary Disease." This and the discussion which followed will be found on pp. 30—35.

Dr. MICHELL CLARKE read a paper embodying observations on the temperature in cases of apoplexy, and on the occasional occurrence of painful œdema, and of loss of the knee-jerk in the paralysed limbs in hemiplegia. This will be printed in full in a future number.

Mr. T. CARWARDINE showed microscopic slides illustrating some points in the pathology of Paget's disease of the nipple, from preparations made by Mr. A. L. Flemming. Enlarged axillary glands were present, and they exhibited secondary deposits of epithelium similar to that in the diseased nipple, although there was no tumour in the true breast tissue. Another specimen showed the epithelial change passing down a galactophorous duct (*a*) as a cellular process passing down the lumen, (*b*) as a proliferation of and change in the cells of the Malpighian layer around the deep part of the duct.—Mr. ROGER WILLIAMS said the history of the evolution of our knowledge of Paget's disease showed at every turn the ill effects entailed by the unscientific use of personal names for the designation of morbid conditions. The result had been such mystification and confusion, that it was necessary before discussing the subject to state precisely what was meant by Paget's disease; for this term had been applied to so many totally different morbid conditions, that it was now seldom used by two persons in the same sense. Paget,[1] who first prominently directed attention to the subject, defined the condition as "disease of the mammary areola preceding cancer of the mammary gland"—an admirable definition which, had it been preserved, would have prevented a flood of misunderstanding and bad practice. It had been repeatedly demonstrated that the areolar disease referred to was nothing but eczema; and the cancer—which developed in the substance of the gland, without there being any obvious connection between the two diseases—was of the acinous *(scirrhus)* variety. Paget admitted that the areolar disease might often be cured, without any ill consequences ensuing; but he thought it was "very often" followed by the development of cancer in the breast. In support of this opinion he adduced no statistical data; for none were then available. Recently the subject had been investigated on a large scale: and it had been shown that the outbreak of mammary cancer had been preceded by eczema of the areola, only in about ½ per cent. of the total cases. We might, therefore, disabuse our minds of the idea that "Paget's disease" played any important part in the pathogeny of mammary cancer. Eczematous affections of the areola were very seldom followed by the development of mammary cancer: hence the breast should never be extirpated for such conditions, unless there were signs of concomitant malignant disease. Thin had described as "Paget's disease" cases of cancer of the mammary ducts (tubular cancer) with secondary inflammation of the nipple and areola by irritant discharges. Another class of cases that had been described as "Paget's disease" were the so-called "eczema cancers": cases in which epithelioma had supervened on eczematous affections of the nipple and areola. Mr. Williams thought the specimen exhibited this evening was an instance of this kind—squamous epithelioma, with dissemination in the axillary glands.

Mr. HARSANT read some notes of a case of actinomycosis occurring in a gentleman living at Taunton, under the care of Dr. Husbands. Six months ago he had put an ear of barley-straw in his mouth. It pricked the mucous membrane under the tongue, causing a painful swelling there which disappeared after a few days. Two or three weeks afterwards a swelling appeared on the front of the neck, over the thyro-hyoid membrane; this had gradually increased until it was as large as a walnut. It presented a semi-fluctuating swelling, the skin over which was red and œdematous. It was diagnosed as being probably actinomycosis, and was freely excised. The wound healed readily and there had been no recurrence. The tumour contained numerous yellow granules, which under the microscope showed the characteristic ray-fungus. Microscopical specimens were shown by Dr. Symes; and a photo-micrograph, prepared by Mr. James Taylor, was thrown on the screen.—Dr. WALDO asked Mr. Harsant if the glands in the neighbourhood of the growth were affected in his case. He considered this a very important point, as the fungus was too large to pass along the lymphatics, and unless pyogenic microbes gained an entrance and set up suppuration the glands remained healthy. The disease spread through the blood-vessels. He remarked that it was

[1] *St. Barth. Hosp. Rep.*, 1874, x. 87.

singular that the bone was nearly always affected when actinomycosis attacked the lower animals, whereas in man it was quite the exception. On the other hand, suppuration was the rule in man and never occurred in animals. He said there were some well authenticated cases in which the internal administration of iodides seemed to cure the patient, and also when an iodide solution was injected into the tissues. Electro-chemical treatment was also well spoken of, the solution being injected and then a current passed through to decompose the substance, and so utilise the antiseptic power of iodine in its nascent state.

Prof. FAWCETT showed through the lantern some sections of the cord, medulla, and midbrain, stained by a modification of the Weigert-Pal method.

J. PAUL BUSH, *Hon. Sec.*

---

